# Data-Driven Models for Objective Grading Improvement of Parkinson’s Disease

**DOI:** 10.1007/s10439-020-02628-4

**Published:** 2020-10-01

**Authors:** Abdul Haleem Butt, Erika Rovini, Hamido Fujita, Carlo Maremmani, Filippo Cavallo

**Affiliations:** 1 The BioRobotics Institute, Scuola Superiore Sant’Anna, Viale Rinaldo Piaggio, 34, 56025 Pontedera, Italy; 2 Department of Excellence in Robotics & AI, Scuola Superiore Sant’Anna, Piazza Martiri della Libertà 33, 56127 Pisa, Italy; 3 Intelligent Software Systems Lab, Iwate Prefectural University, 152-52 Sugo, Takizawa, Iwate Japan; 4 U.O. Neurologia, Ospedale delle Apuane (AUSL Toscana Nord Ovest), Viale Mattei 21, 54100 Massa, Italy; 5 The Department of Industrial Engineering, University of Florence, Via Santa Marta 3, 50139 Florence, Italy; 6 The Creative Technology Department, Faculty of Computing and Artificial Intelligence, Air University Islamabad Pakistan, Service Road E-9/E-8, Islamabad, Pakistan

**Keywords:** ANFIS, Artificial intelligence, Regression models, Predictive methods, Parkinson disease severity

## Abstract

Parkinson’s disease (PD) is a progressive disorder of the central nervous system that causes motor dysfunctions in affected patients. Objective assessment of symptoms can support neurologists in fine evaluations, improving patients’ quality of care. Herein, this study aimed to develop data-driven models based on regression algorithms to investigate the potential of kinematic features to predict PD severity levels. Sixty-four patients with PD (PwPD) and 50 healthy subjects of control (HC) were asked to perform 13 motor tasks from the MDS-UPDRS III while wearing wearable inertial sensors. Simultaneously, the clinician provided the evaluation of the tasks based on the MDS-UPDRS scores. One hundred-ninety kinematic features were extracted from the inertial motor data. Data processing and statistical analysis identified a set of parameters able to distinguish between HC and PwPD. Then, multiple feature selection methods allowed selecting the best subset of parameters for obtaining the greatest accuracy when used as input for several predicting regression algorithms. The maximum correlation coefficient, equal to 0.814, was obtained with the adaptive neuro-fuzzy inference system (ANFIS). Therefore, this predictive model could be useful as a decision support system for a reliable objective assessment of PD severity levels based on motion performance, improving patients monitoring over time.

**Electronic supplementary material:**

The online version of this article (10.1007/s10439-020-02628-4) contains supplementary material, which is available to authorized users.

## Introduction

Parkinson’s disease (PD) is a common degenerative disorder of the central nervous system characterized by both motor and non-motor symptoms. Traditionally, in clinical practice, PD motor signs are assessed by neurologists while they observe patients performing motor tasks described in section III of the Movement Disorders Society Unified Parkinson’s Disease Rating Scale (MDS-UPDRS III) and patient diaries.^10^

Thus, the clinical evaluation is mainly based on the experience of clinicians that assign a score ranging from 0 (no signs clinically evident) to 4 (severely impaired) for each task performed by patients. Finally, the overall score, which is the sum of all the exercises, represents a semi-quantitative index for identifying the severity of the impairments due to the pathology. To evaluate the PD progression, it is necessary to understand the long-term monitoring of the disease, assessing the patients periodically.

This traditional evaluation, based on clinical scales, however, relies on clinical expertise, and is subjected to inter- and intra- observer variability.^20^ Moreover, using diaries can be difficult for patients with recalling bias when reporting motor fluctuations. Therefore, the traditional evaluation methods are suboptimal for PD diagnosis and monitoring, and novel methods and technologies should be investigated.^6^,^22^

In this direction, different tools have investigated as diagnostic or prognostic indicators, such as: the use of image guidance and robot-assisted techniques to improve the accuracy of electrode placement during deep brain stimulation surgery^29^; the use of Leap Motion Controller as non-invasive methods to assess motor tasks^4^; the use of EEG algorithmic complexity as prognosis for idiopathic rapid eye movement sleep behaviour disorder (RBD) which is a serious risk factor for PD^23^; or the use of an olfactory identification test as prognosis for idiopathic hyposmia that is another preclinical marker of PD.^16^ Furthermore, recent studies endorse the idea that wearable devices together with artificial intelligence technology could provide decision-making support systems that can help the clinical practitioners in the objective assessment of PD,^26^ allowing also the long-term monitoring and management of the pathology.^18^ Furthermore, accurately defining PD subtypes can be challenging as well. Since PD is a progressive disease with heterogeneity in individual disease trajectories,^31^ investigating the longitudinal clinical records is necessary to understand the disease progression. To detect the motor impairment changes and to identify PD related symptoms in individual, multiple sensor-based measurements of UPDRS could provide useful information.^8^ Capturing the data from multiple affected limbs (upper and lower) provides an opportunity for investigating the relationships between clinical rating scale of motor tasks. Moreover, machine learning methods could provide a useful decision support system to support clinicians in PD management.^6^,^22^

In this direction, recent studies analysed motor tasks for PD subtyping identification with wearable technology that primarily relied on machine learning techniques^9^ to develop predictive models. This is basically treated as a clustering problem, where the model can compute continuous numeric signal metrics for the subgrouping of the clusters. In previous studies, for the assessment of the motor dysfunction in PD, different regression models were applied. The regression analysis provides a functional relationship among variables that are expressed in the form of a dependent variable and one or more explanatory variables. In a recent study, logistic regression was used to classify freezing of gait and non-freezing of gait in PD patients affected by motor fluctuations by using acceleration sensors.^19^ Regression techniques were used to assess the fine motor skills of PD patients and healthy subjects through the analysis of touchscreen typing pattern.^13^ In this study, evolving the binary classification problem into a regression analysis for estimating the severity of individual PD motor symptoms could assist physicians by providing explainable insights into the subject’s condition against PD. Furthermore, a support vector machine regression model was also successfully used for remote tracking of PD based on speech signal analysis.^7^ Additionally, Patel *et al*.^18^ proposed to implement a regression random forest for the longitudinal assessment of motor fluctuations by analysing MDS-UPDRS III tasks from both upper and lower limbs with wearable sensors. Finally, a treatment-response index estimated from wearable sensors for quantifying PD motor states was developed by Thomas *et al*.,^27^ thus supporting personalized treatment for advanced PD patients.

In general, very few studies, to the best of our knowledge, use the clinical score as a predictor against the extracted parameters from wearable technology and apply regression models to classify healthy and PD subjects. Moreover, there are few studies focused on multiple sensor-based measurements approach to capturing data from multiple affected limbs of PD. First, our study aims to understand the significance of the extracted parameters for the prediction of Parkinson disease progression using motion data acquired from two wearable devices, called SensHand^6^ and SensFoot.^22^ In this study, we aimed at investigating the relationship between the extracted information from the sensors and the clinical scores provided by the clinicians according to the MDS-UPDRS. To achieve this goal, multiple feature selection methods,^4^,^15^ combined with multiple regression models,^11^,^12^,^17^,^25^ were investigated to understand which technique could be the most helpful for longitudinal clinical data analysis and long-term monitoring of PD progression. We already investigated classification approach with supervised learning in previous works, considering both binary classification^21^ (i.e., PD patients vs healthy controls), and multigroup classification (i.e., PD patients, healthy subjects, and subjects with idiopathic hyposmia that are at risk for developing the disease).^6^,^22^ Differently, here we want to investigate whether our system is able to identify the pathology progression, from the mild to advanced stages. Therefore, we applied the regression approach, which is projected towards the possibility to have a continuous curve of the evolution of the pathology for improving the fine assessment of PD patients according to their motor performances.

The remain of the paper is structured as follows: in the Materials and Methods section we describe the participants to the study, the wearable system used to measure the motor performance, the experimental protocol and data analysis. In the Results section, the findings about features selection and the accuracy of the regression models are reported. Then, in Discussion section, we provide an interpretation of the results considering also the limitations of the study.

## Materials and Methods

**Figure 1:**
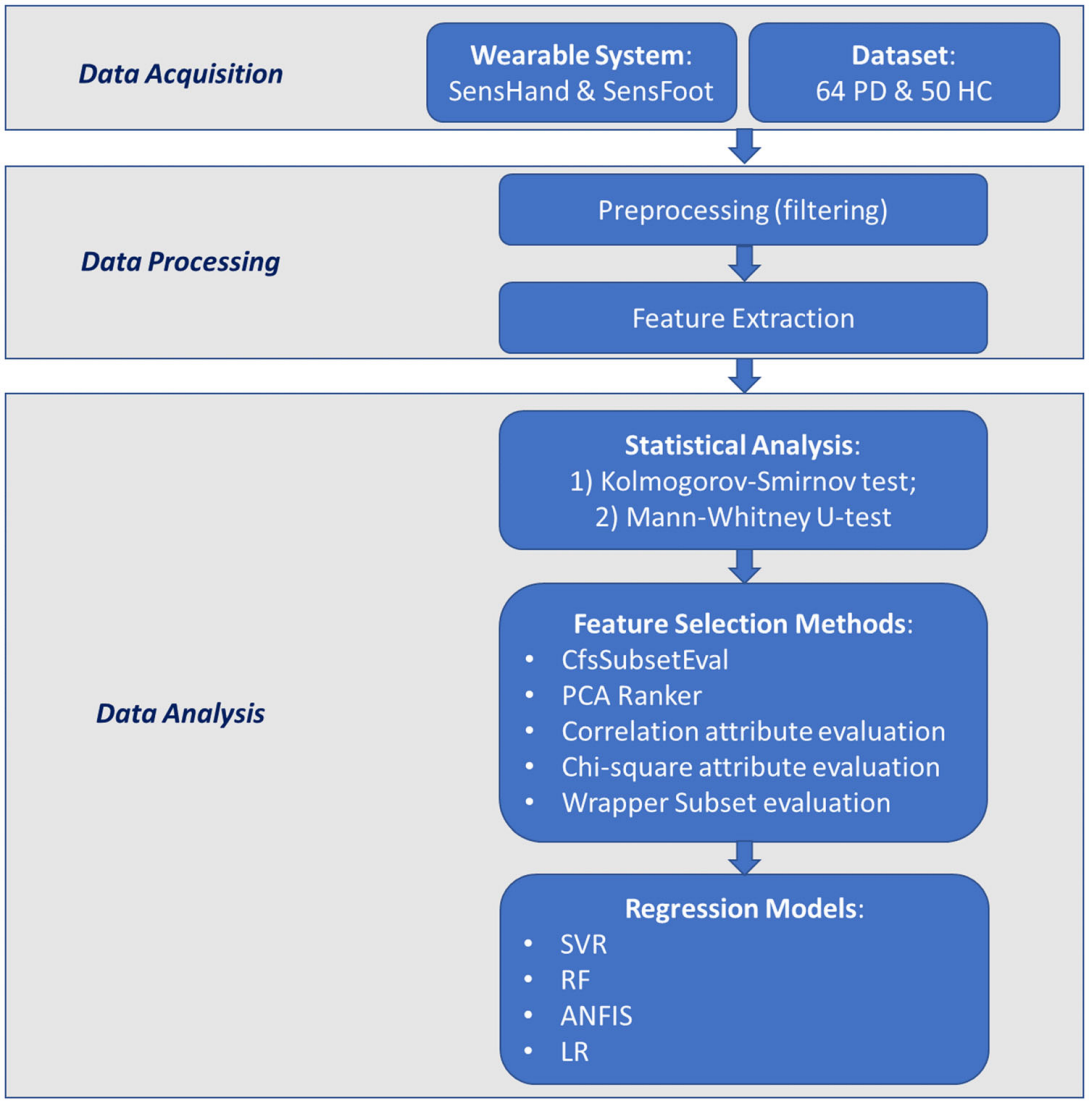
Methodology flowchart.

The methodology applied in this study is synthesized in the flowchart in Fig. 1.

### Involved Subjects

A total of 114 subjects (64 PD patients: 40 males, 24 females; mean age ± standard deviation (SD) 66.60 ± 8.87 years old, mean Hoehn&Yahr stage ± SD 1.9 ± 0.7, mean MDS-UPDRS III score ± SD 15.7 ± 8.9; and 50 HC: 39 male, 11 females; mean age ± standard deviation 65.46 ± 2.70 years old) were involved in this study. The neurologist that supported the experiment defined the exclusion criteria. Those who had impairments (e.g., prosthesis or arthrosis) or diseases other than PD (e.g., neurological) that could affect the performance of the required tasks were excluded from the study. Before and during the experiments, all patients were on the medication state and were examined by the neurologist. All subjects involved in this study gave written informed consent before the beginning of the experimental sessions. Procedures of this study were approved by the local Medical Ethical Committee (Azienda Sanitaria Locale, Massa and Carrara, Italy; Approval No. 1148/12.10.10).

### Instruments

**Figure 2:**
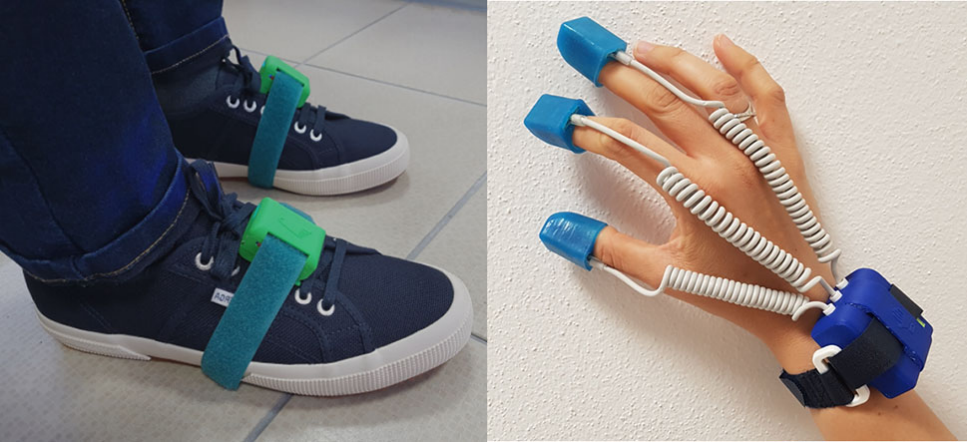
The wearable system: SensHand and SensFoot.

The system used in this work is composed of two wearable modules that provide an objective and quantitative analysis of the movements of the upper and lower limbs through inertial measurement units (IMUs). IMUs, integrated into the iNEMO-M1 board and composed of three-axis gyroscope L3G4200D, six-axis geomagnetic module LSM303DLHC and ARM-based 32-bit microcontroller STM32F103RE (STMicroelectronics, Italy), were used to develop SensHand and SensFoot device (Fig. 2), respectively, for upper and lower limb analysis.^22^

The SensHand includes 4 iNEMO-M1, which are positioned on the wrist and on the distal phalanges of the thumb, index, and middle fingers and connected and synchronized between them through the CAN-bus standard.

The SensFoot includes a iNEMO-M1 and is placed on the dorsum of the subject’s foot with a Velcro-strap to ensure integrity between the foot and sensor.^6^ Both modules are supplied by a rechargeable LiPo battery and integrated with a Bluetooth module (SPBT2632C2A, v3.0, STMicroelectronics) for wireless data transmission to a remote PC, where data were stored and analysed offline. Both modules collected data with a sampling frequency of 100 Hz.

### Experimental Protocol and Feature Extraction

The trial session included exercises for upper and lower limbs, based on the tasks of the MDS-UPDRS III. During the exercises, the subjects took a comfortable and standardized sitting position, holding a right angle between the trunk and the thigh (on the hip) and between the thigh and the shin (on the knee). Initial and final fixed positions were established for each exercise to allow a static acquisition of 3 s to acquire the initial position as a reference for each trial. Participants were directed to perform each exercise for 10 s, as fast as possible. The following are descriptions of the 13 exercises (see Supplementary Material for an explicative video about tasks performance):

- Gait: The subject stood still with arms along sides, and then linearly walked 15 meters. The subject had to walk at their preferred speed in the most natural way (MDS-UPDRS 3.10).
- Rotation (ROTA): At the beginning and at the end of the cycle, the subject had to stand still with arms along sides. The subject turned 360°. The rotation was performed both in the clockwise and counterclockwise directions (MDS-UPDRS 3.10).
- Heel tapping, toe pin (HTTP): The subject touched the heel on the floor, and the forefoot was always in contact with the ground.
- Toe tapping, heel pin (TTHP): The subject touched the toe on the floor and kept the heel in contact with the ground (MDS-UPDRS 3.7).
- Heel-toe tapping (HETO): The subject tapped the heel and forefoot on the floor alternately.
- Heel–heel tapping (HEHE): The subject touched the heel on the floor and kept the forefoot up. (MDS-UPDRS 3.9).
- Forearm pronation-supination (PSUP): The subject placed the arm in front, with the wrist stable, the hand prone. The movements of the prono-supination had to be carried out in parallel with the floor (MDS-UPDRS 3.6).
- Hand opening–closing (OPCL): The subject bent the arm at the elbow, which was resting on the table, and held the hand palm up in front. The subject had to open and close the hand alternately, holding the forearm and wrist stable (MDS-UPDRS 3.5).
- Thumb-forefinger tapping (THFF): The subject held the hand on the desk so that the plane where thumb and forefinger tapped was parallel to the table. The thumb and forefinger were in contact in the starting position; then, the subject touched the forefinger against the thumb (MDS-UPDRS 3.4).
- Thumb-middle finger tapping (THMF): The subject kept the hand fixed on the desk so that the plane where the thumb and middle finger joined was parallel to the table. The thumb and middle finger were in contact in the starting position; then, the subject touched the middle finger to the thumb.
- Arms swing (ARMS): The subject walked in a linear manner for 15 meters at the preferred speed (MDS-UPDRS 3.10).
- Rest tremor (HRST): The subject placed the hand on the table. For the entire duration of the exercise, the subject kept the hand completely relaxed, without contrasting the potential tremor (MDS-UPDRS 3.17).
- Postural tremor (POST): The subject placed the arm in front with a stable wrist, the hand in a prone position and the fingers about 1 cm apart. For the entire duration of the exercise, the subject remained in this fixed position (MDS-UPDRS 3.15).

Data acquired from both accelerometer and gyroscope were preprocessed with adequate filters: bandpass 0.5–15 Hz for HRST, bandpass 0.5–20 Hz for POST, low-pass with cutoff frequency equals to 3 Hz for GAIT, low-pass with cutoff frequency equals to 5 Hz for all the others. Custom-made algorithms were applied to calculate spatiotemporal and frequency features, as already described in previous works 6,22 for both upper and lower limbs, obtaining 190 parameters.

All exercises were performed twice, both for the left and right sides; then, the average value of the parameters for each side was calculated and used for the data analysis.

### Data Analysis and Machine Learning Methods

#### Statistical Analysis

All the statistical analyses were performed with SPSS21 (IBM, Armonk, North Castle, NY, USA). First, the Kolmogorov–Smirnov test was applied to each measured parameter to verify if it had a normal or abnormal distribution. Since each parameter had an abnormal distribution, the unpaired Mann–Whitney (MW) U-test for non-parametric samples was calculated^28^ to identify the features that were statistically significant for distinguishing between the HC and PD groups. The test is issued to determine whether two independent samples (in our case healthy and patients) have the same distributions with the same median without assuming normal distributions.^9^ The choice of the cut-off points to accept the alternative hypothesis was *p* ≤ 0.05.^2^ All the measured features were normalized according to the linear relationship reported in Eq. (1):

1 $$x^{\prime} = \frac{x - \hbox{min} \left( x \right)}{\hbox{max} \left( x \right) - \hbox{min} \left( x \right)}$$

The normalized features were used for further investigations in this paper. This scaling procedure is more robust to control for both scale (min and max) and location (mean). The mean and standard deviation of a group gives information on how different the individual group population is from the other group in terms of the location of numeric values (mean) and variation in the values (standard deviation). With the linear scaling, it is easier to estimate the group difference in terms of mean and standard deviation between the values 0 and 1.

Feature Selection Algorithms All the features identified as significant by the MW test were included for the analysis with multiple feature selection methods. The feature selection step involves finding the most relevant subset of features from the original set by eliminating inappropriate or redundant features.^14^ In this study, multiple tests were run using various algorithms developed in Weka 3.8 to define the optimal subsets of attributes, which would help to avoid over-fitting due to redundant information. The chosen feature selection methods have been used in previous studies to obtain the optimum set of parameters to increase the overall accuracy of the system.^4^,^15^

Specifically, five feature selection methods^1^ were investigated in this study:

- Correlation-based feature subset evaluation (CfsSubsetEval): This method evaluates the worth of a subset of attributes that work well together, preferring sets of attributes that are highly correlated with the class but that have low intercorrelation. This multivariate method ignores irrelevant and redundant features from the dataset. This evaluator is implemented with the Best-First search algorithm.
- PCA ranker: Ranker methods basically use single- attribute evaluators to generate a ranked list. Unlike other single-attribute evaluators, PCA transforms the set of attributes and then ranks the new attributes according to their eigenvalues.
- Correlation attribute evaluation: This method evaluates the worth of an attribute by measuring the correlation coefficient, based on Pearson’s correlation, between each attribute and the class.
- *Χ*^2^ attribute evaluation: The *Χ*^2^ is calculated between each feature and the target, and the features with the best *Χ*^2^ scores are selected. This method determines if the association between two categorical variables of the sample would reflect their real association in the population.
- Wrapper subset evaluation: This method evaluates the worth of the attribute sets by using a learning scheme, and it employs cross-validation to estimate the accuracy of the learning scheme for each set of attributes. Based on the prediction performance, a score is assigned to each subset, and the best subset is chosen.

### Regression Methods for PD Severity Evaluation

After selecting the optimal feature array, multiple regression techniques were applied to evaluate the accuracy of the proposed system in automatically quantifying the severity of the disease in clinical terms. Indeed, the regression models can estimate the functional relationship between explanatory variables and a target variable. Moreover, regression trees can be studied to develop a prediction model able to overcome the discrete scores and severity levels of the traditional clinical scales toward the possibility to associate a continuous response score to each patient according to the pathology progression. In particular, supervised machine learning techniques, such as support vector regression (SVR),^17^ random forest (RF),^3^ adaptive neuro-fuzzy inference system (ANFIS),^12^,^17^ and linear regression (LR)^11^ were tested in this work. Based on preliminary investigations with different hyperparameters configurations, default hyperparameters tuning was used since it allowed obtaining the best accuracy in regression models. All the methods were run over the ten-fold cross-validation. For applying the regression models, both the clinical scores and the extracted features from the sensors were used in the normalized form (see Eq. 1) to make sure to fit the model completely and not to overlap the predictor and target values. All the prediction algorithms were developed on the open-source platform Weka 3.8 except for the ANFIS model, which was developed on MATLAB 2019b (The MathWorks, Inc., Natick, MA, USA). Following, a description of each model is reported:

### Support Vector Regression

Support vector machine was proposed for solving the binary classification problem. As for the multi-class classification problem, the form of SVR allows for estimating the continuous function of training datasets. It is able to model complex nonlinear relationships by using an appropriate kernel function that maps the input into higher-dimensional feature space and transforms the nonlinear relationships into linear forms.^17^ Since previous studies endorsed the significance of the RBF kernel,^11^ it was used also in this work. For the prediction, the margin of tolerance (epsilon) for the loss function is set to 0.1, while the weight vector is set to measure the number of successive trails.

SVR looks for the optimal function $f_{x} = \left( {w,x} \right) + b$, where w is the weight vector and b is the threshold value. Thus, SVR minimizes the expected risk prediction. The optimal function is solved in the feature space to make predetermined risk function minimization. After the introduction of the kernel, the regressive function becomes as in Eq. (2):

2 $$f_{x} = \mathop \sum \limits_{i = 1}^{n} \left( {\alpha_{i} - \alpha_{i}^{*} } \right)K\left( {x_{i} ,x} \right) + b$$

where $K\left( {x_{i} ,x} \right)$ is a kernel function, and $\alpha_{i}$ and $\alpha_{i}^{ *}$ are Lagrangian multipliers.^17^

### Adaptive Neuro-Fuzzy Inference System

The path for building fuzzy systems and information extraction is usually based on two parts (i.e., the knowledge formulation from the conscious path and knowledge formulation from the subconscious path). In the first path, rules and membership functions (MF) are consequent from human intelligence based on their expertise, experience, and understanding. With the subconscious formulation of knowledge, rules and membership functions are developed using automated techniques such as gradient methods, learning techniques, and clustering methods. Fuzzy reasoning is a procedure to derive a conclusion from a set of fuzzy *if*–*then* rules. These rules are evaluated using the MF, which is a curve where each point in the input space maps the degree of membership between 0 and 1. The fuzzification step maps the input characteristics to quantify the grade of membership of the fuzzy set *via* the Gaussian MF with hybrid learning methodology. Then, defuzzification converts the input MF into *if*–*then* rules, which derive the output characteristics for output MFs. Before gathering data using a fuzzy C-means (FCM), some parameters need to be quantified, such as: number of clusters (in our case 2), partition matrix exponent (default is 2) for final partition or membership function matrix U, maximum number of repetitions (default is 200), and minimum improvement (default is 1e-5). For this work, the default stopping threshold of $10^{ - 5}$ was applied.^24^ The final step is associated with the sum of all the output MFs into one single-value output. In this study, a fuzzy inference system (FIS) was generated with an FCM algorithm to cluster the inputs. The dataset was divided into a predefined number of clusters using the FCM algorithm. The number of clusters is pre-specified to help in defining the preliminary number of rules for each cluster and the membership of each set of data in the cluster. This technique is also known as semi-supervised since the information about the MF is predefined by the user with the identifying hyper-parameters.^17^

### Random Forest

Random forest uses multiple learning algorithms for forecasting both classification and regression problems. RF combines the results of decision trees trained by the “bagging” method. RF is one of the most accurate classifiers among the current algorithms that use decision tree methods and maintains accuracy when a large proportion of the data are missing. It can handle many input variables. It generates an internal unbiased estimation of the generalization error as the forest building progresses.. It runs quickly to produce a forest of decision trees for the classifier. It is assumed that the number of cases in the training set is N, and the number of variables in the classifier is M. The system selects the number of input variables that will be used to determine the decision at a node of the tree. For each node of the tree, m of the M variables is randomly selected, and the decision at the node is based on them. The best split based on these m variables is calculated in the training set.

### Linear Regression

Linear regression is a linear approach to modelling the relation between scalar response and one or more explanatory variables. When the outcome and all the attributes are numeric, linear regression is a natural technique to consider. The idea is to express the outcome as a linear combination of the attributes, with predetermined weights as in Eq. (3):

3 $$x = w_{0} + w_{1} a_{1} + w_{2} a_{2} + \cdots w_{k} a_{k}$$

where *x* is the class; $a_{1} ,a_{2} \ldots .a_{k}$ are the attribute values; and $w_{0} ,w_{1} , \ldots w_{k}$ are weights. The weights are calculated from the training data.^30^

## Results

### Mann–Whitney Significance Test Between PD Patients and HC

The unpaired MW U-test was employed to identify the significant features, among the 190 extracted parameters, for comparing healthy controls and the PD group. Both right and left sides were separately compared, and a parameter was considered significant if the MW U-test showed statistical significance in at least one side. Normalized mean values, standard deviations and *p* value were reported in Tables 1 and 2 for each measured feature. Statistically significant parameters were selected from all the exercises; in particular, 52 features were selected from the lower limbs and 78 from the upper limbs. Therefore, each exercise contributed to distinguishing PD and HC. Furthermore, both features extracted from accelerometer and gyroscope showed significance, thus suggesting that both the sensors are equally important for the assessment of the disease progression.

**Table 1:** Extracted parameters (mean ± standard deviation) as normalized values from lower limb exercises for both PD patients and HC subjects.

Ex	Biomechanical features	Acronym	Patients	Control	*p* value
GAIT	Gait Time Gait Strides Gait Frequency Stride Time Swing Time Stance Time Relative Stance Angular Excursion	F_GT F_GSTRD F_GF F_GSTRDT F_GSWT F_GSTT F_GRS F_GANG	0.155 ± 0.145 0.127 ± 0.142 0.544 ± 0.222 0.127 ± 0.142 0.329 ± 0.144 0.379 ± 0.202 0.564 ± 0.183 0.671 ± 0.191	0.928 ± 0.426 0.661 ± 0.363 0.571 ± 0.169 0.661 ± 0.363 0.311 ± 0.104 0.385 ± 0.154 0.594 ± 0.136 0.746 ± 0.125	0.002* 0.000* 0.803 0.000* 0.380 0.623 0.417 0.016*
ROTA	Rotation Time Rotation Strides Rotation Frequency Stance Time Relative Stance	F_RT F_RSTRD F_RF F_RSTT F_RRS	0.226 ± 0.548 0.207 ± 0.176 0.372 ± 0.211 0.207 ± 0.176 0.596 ± 0.150	0.787 ± 0.548 0.104 ± 0.503 0.437 ± 0.248 0.104 ± 0.503 0.552 ± 0.142	0.000* 0.000* 0.220 0.000* 0.127
HTTP	Heel Frequency Taps Heel angle Heel Frequency CV Heel angle CV Energy Expenditure	F_HTTP_fH F_HTTP_taps F_HTTP_H-angle F_HTTP_CV-fH F_HTTP_CV-Hangle F_HTTP_IAV	0.521 ± 0.220 0.514 ± 0.221 0.233 ± 0.199 0.539 ± 0.248 0.660 ± 0.260 0.552 ± 0.198	0.691 ± 0.140 0.685 ± 0.143 0.345 ± 0.215 0.507 ± 0.202 0.651 ± 0.235 0.706 ± 0.105	0.000* 0.000* 0.007* 0.466 0.985 0.000*
TTHP	Toe Frequency Taps Toe angle Toe Frequency CV Toe angle CV Energy Expenditure	F_TTHP_fT F_TTHP_taps F_TTHP_T-angle F_TTHP_CV-fT F_TTHP_CV-Tangle F_TTHP_IAV	0.490 ± 0.212 0.532 ± 0.201 0.634 ± 0.226 0.496 ± 0.259 0.634 ± 0.226 0.670 ± 0.244	0.530 ± 0.194 0.581 ± 0.178 0.420 ± 0.253 0.425 ± 0.259 0.528 ± 0.283 0.820 ± 0.580	0.128 0.118 0.021* 0.215 0.063 0.020*
HETO	Toe Frequency Heel Frequency Heel-Toe Frequency Taps Toe Angle Heel Angle Heel-Toe Frequency CV Toe Angle CV Heel Angle CV Energy Expenditure	F_HETO_fTT F_HETO_fHH F_HETO_fHT F_HETO_taps F_HETO_ToeAng F_HETO_HeelAg F_HETO_fHT_Cv F_HETO_ToeAngC F_HETO_HeelAng_CV F_HETO_IAV	0.308 ± 0.117 0.312 ± 0.117 0.315 ± 0.121 0.298 ± 0.121 0.286 ± 0.160 0.285 ± 0.151 0.330 ± 0.126 0.286 ± 0.160 0.285 ± 0.151 0.762 ± 0.182	0.420 ± 0.155 0.425 ± 0.154 0.426 ± 0.146 0.416 ± 0.156 0.432 ± 0.173 0.422 ± 0.169 0.370 ± 0.154 0.432 ± 0.173 0.422 ± 0.169 0.855 ± 0.076	0.000* 0.000* 0.000* 0.000* 0.000* 0.000* 0.152 0.000* 0.000* 0.007*
HEHE	Signal Average Power Fundamental Frequency Peak in PSD Energy Expenditure	F_HEHE_AVGPWR F_HEHE_FREQ F_HEHE_PEAK F_HEHE_IAV	0.202 ± 0.229 0.282 ± 0.140 0.121 ± 0.139 0.221 ± 0.167	0.525 ± 0.202 0.330 ± 0.115 0.339 ± 0.196 0.441 ± 0.167	0.000* 0.028* 0.000* 0.000*

Also, *p* values from the Mann–Whitney test are reported

*Statistical significance at 95% confidence level (*p* < 0.05)

**Table 2:** Extracted parameters (mean ± standard deviation) as normalized values from upper limb exercises for both PD patients and HC subjects.

Ex	Biomechanical features	Acronym	Patients	Control	*p* value
Hand opening/closing (OPCL)	Movement Frequency Movements Movement amplitude Opening velocity Closing velocity Variability in frequency Variability in amplitude Energy expenditure	H_fOC H_numOC H_excOC H_wop H_wcl H_fCV-OC H_tetaCV-OC H_OC-IAV	0.398 ± 0.197 0.409 ± 0.201 0.466 ± 0.230 0.412 ± 0.221 0.373 ± 0.213 0.324 ± 0.217 0.526 ± 0.260 0.305 ± 0.258	0.570 ± 0.139 0.580 ± 0.139 0.425 ± 0.176 0.538 ± 0.201 0.544 ± 0.190 0.222 ± 0.124 0.502 ± 0.194 0.555 ± 0.209	0.000* 0.000* 0.449 0.004* 0.000* 0.033 0.877 0.000*
Forearm prono/supination (PSUP)	Movement Frequency Movements Movement amplitude Supination velocity Pronation velocity Variability in frequency Variability in amplitude Energy expenditure	H_fPS H_numPS H_excPS H_wps H_wsp H_fCV-PS H_tetaCV-PS H_PS-IAV	0.399 ± 0.252 0.415 ± 0.255 0.409 ± 0.180 0.350 ± 0.202 0.340 ± 0.131 0.239 ± 0.198 0.305 ± 0.195 0.204 ± 0.173	0.505 ± 0.181 0.526 ± 0.179 0.634 ± 0.157 0.6515 ± 0.15 0.590 ± 0.165 0.214 ± 0.173 0.237 ± 0.189 0.468 ± 0.233	0.011* 0.009* 0.000* 0.000* 0.000* 0.502 0.015* 0.000*
Thumb-forefinger tapping (THFF)	Movement Frequency Movements Movement amplitude Opening velocity Closing velocity Variability in frequency Variability in amplitude Energy expenditure	H_fTF H_tapTF H_tetaTF H_woTF H_wcTF H_fCV-TF H_tetaCV-TF H_TF-IAV	0.575 ± 0.219 0.562 ± 0.223 0.216 ± 0.205 0.297 ± 0.244 0.297 ± 0.243 0.436 ± 0.252 0.647 ± 0.280 0.324 ± 0.192	0.741 ± 0.151 0.738 ± 0.149 0.716 ± 0.129 0.322 ± 0.202 0.336 ± 0.201 0.285 ± 0.186 0.619 ± 0.233 0.436 ± 0.165	0.000* 0.000* 0.481 0.689 0.437 0.006* 0.689 0.006*
Thumb-middle finger tapping (THMF)	Tap Frequency Movements Movement amplitude Opening velocity Closing velocity Variability in frequency Variability in amplitude Energy expenditure	H_fTM H_tapTM H_tetaTM H_woTM H_wcTM H_fCV-TM H_tetaCV-TM H_TM-IAV	0.910 ± 0.414 0.498 ± 0.234 0.159 ± 0.146 0.308 ± 0.239 0.292 ± 0.234 0.491 ± 0.254 0.626 ± 0.259 0.605 ± 0.129	0.127 ± 0.262 0.700 ± 0.152 0.134 ± 0.928 0.431 ± 0.214 0.431 ± 0.209 0.270 ± 0.214 0.532 ± 0.245 0.737 ± 0.107	0.000* 0.000* 0.612 0.010 0.003* 0.000* 0.087 0.000*
Arms swing arms swinging (ARMS)	Swing arms frequency Movements Movement amplitude Front velocity Back velocity Variability in frequency Variability in amplitude Energy expenditure	H_fSW H_swing H_tetaSW H_wfSW H_wbSW H_fCV-SW H_tetaCV-SW H_SW-IAV	0.410 ± 0.133 0.304 ± 0.142 0.261 ± 0.189 0.289 ± 0.181 0.467 ± 0.260 0.343 ± 0.307 0.322 ± 0.223 0.180 ± 0.146	0.426 ± 0.687 0.250 ± 0.611 0.520 ± 0.223 0.492 ± 0.230 0.411 ± 0.268 0.215 ± 0.214 0.534 ± 0.206 0.152 ± 0.666	0.486 0.025 0.000* 0.000* 0.253 0.029 0.000* 0.347
Postural tremor (POST)	Accelerometer signal power Acc fundamental freq. Acc %power band [3.5-7.5 Hz] Acc %power band [8-12 Hz] Energy expenditure Gyroscope signal power Gyr fundamental frequency Gyr %power in band [3.5-7.5 Hz] Gyr %power in band [8-12 Hz]	H_a_pwrP H_a_fP H_a_pwrpP1 H_a_pwrpP2 H_IAV-P H_g_pwrP H_g_fP H_g_pwrpP1 H_g_pwrpP2	0.334 ± 0.215 0.556 ± 0.168 0.334 ± 0.215 0.412 ± 0.209 0.085 ± 0.188 0.040 ± 0.190 0.483 ± 0.215 0.311 ± 0.230 0.354 ± 0.206	0.183 ± 0.111 0.591 ± 0.235 0.183 ± 0.111 0.501 ± 0.208 0.034 ± 0.014 0.000 ± 0.000 0.535 ± 0.271 0.159 ± 0.697 0.430 ± 0.144	0.000* 0.342 0.000* 0.078 0.002* 0.007* 0.276 0.000* 0.013*
Rest tremor (REST)	Accelerometer signal power Acc fundamental frequency Acc %power in band [3.5-7.5 Hz] Energy expenditure Gyroscope signal power Gyr fundamental frequency Gyr %power in band [3.5-7.5 Hz]	H_a_pwrR H_a_fR H_a_pwrpR2 H_IAV-R H_g_pwrR H_g_fR H_g_pwrpR2	0.340 ± 0.189 0.525 ± 0.246 0.244 ± 0.121 0.432 ± 0.286 0.521 ± 0.149 0.508 ± 0.213 0.435 ± 0.219	0.437 ± 0.210 0.542 ± 0.220 0.193 ± 0.651 0.261 ± 0.132 0.006 ± 0.116 0.512 ± 0.222 0.288 ± 0.115	0.041* 0.854 0.001* 0.004* 0.000* 0.948 0.000*

Also, *p* values from the Mann–Whitney test are reported

*Statistical significance at 95% confidence level (*p* < 0.05)

One hundred thirty significant parameters were selected and further investigated with the feature selection methods to select the best subset of features that could be applied as input to data-driven predictive models (i.e., SVR, RF, ANFIS, and LR) to improve the PD severity classification.

### Predictive Accuracy from the Regression Methods

The four investigated regression algorithms were applied to each of the five feature selection methods, and the accuracy of the obtained results are reported in terms of correlation coefficient and root mean squared error in Table 3.

**Table 3:** Predictive accuracy of the evaluated regression methods reported as correlation coefficients using different selection feature methods.

Feature selection method	Selected features	Regression method			
		SVR	RF	ANFIS	LR
CfsSubsetEval Best first search	FL_GT; FL_GSTRD; FR_HETO_taps; HR_excPS; HR_PS-IAV; HL_fPS; HL_PS-IAV; HL_TF-IAV; HR_tapTM; HR_woTM; HL_tetaCV-SW; HR_IAV-P; HR_g_pwrpP1	0.799 RMSE = 0.117	0.790 RMSE = 0.121	0.814 RMSE = 0.101	0.738 RMSE = 0.135
PCA ranker	Principal Components (PC1 – PC19)	0.596 RMSE = 0.158	0.658 RMSE = 0.1506	0.385 RMSE = 0.217	0.497 RMSE = 0.181
Correlation attribute evaluation	FR_GSTRD;FL_GT; FR_RSTRD;FR_RT; FL_RSTRD;FR_GANG; HR_tapTM; HL_tapTF; HR_wfSW; FL_HETO_ToeAng; HL_a_pwrR; HL_g_pwrpR2; HR_IAV-P; HR_fCV-TF; HL_a_pwrpP1	0.5937 Rmse = 0.158	0.648 Rmse = 0.146	0.517 Rmse = 0.1905	0.406 Rmse = 0.199
*Χ*^2^ attribute evaluation	FR_GT; FR_GSTRD; FR_GANG; FL_GT; FR_RT; FR_RSTRD; FL_RT, FL_RSTRD; FL_HETO_ToeAng; HL_TapTF; HR_tapTM; HL_tapTM; HR_wfSW; HL_g_pwrpP1; HL_g_pwrpR2	0.6244 RMSE = 0.1539	0.640 RMSE = 0.149	0.652 RMSE = 0.1999	0.475 RMSE = 0.149
Wrapper subset evaluation	FR_GT; FR_GSTRD; FR_GANG; FL_GT; FR_RSTRD; FL_RT; FL_RSTRD; FL_HETO_ToeAng; HL_tapTF; HR_tapTM; HL_tapTM; HL_a_PwrR	0.6057 RMSE = 0.1569	0.606 RMSE = 0.1569	0.423 RMSE = 0.2262	0.400 RMSE = 0.1981

The correlation-based feature subset evaluation feature selection method with the Best-First search algorithm selected thirteen featuresas input for the predictive models. The best-performing model was ANFIS with 0.814 accuracy (as correlation coefficient), whereas SVR, RF, and LR, obtained 0.799, 0.790, and 0.738, respectively.

Nineteen parameters were selected with the PCA ranker method, 20 parameters with the correlation attribute evaluation, 15 parameters with the *Χ*^2^ attribute evaluation, and 12 with the wrapper method. RF resulted in the best performance for PCA ranker (0.685), correlation attribute (0.648), and wrapper evaluation (0.606). Differently, ANFIS achieved the best performance for the aforementioned *CfsSubsetEval* and for the *Χ*^2^ attribute evaluation method.

## Discussion

This study aimed at providing an objective assessment of fine motion performance decline in PD motor symptoms and at examining different regression models that allowed the identification of a linear relationship between the measured motion parameters and the clinical scores assigned by the neurologists according to the MDS-UPDRS III. Currently, indeed, the clinical scales represent the gold standard for clinical evaluations. Therefore, the first step of our study is to demonstrate that our system agrees with traditional clinical evaluations. Furthermore, it offers the neurologist the opportunity to objectify the clinical assessment. This is a fundamental step to achieve the trust of the neurologist in adopting our system as a decision support tool for improving PD diagnosis procedure. Using regression algorithms can significantly improve the method of performing PD diagnosis because they not only allow identification of whether or not a subject has motor impairments caused by the pathology, as supervised binary classification algorithms can be used, but they also could allow better ranking of the disease progression. Regression models, indeed, permit quantification of the grade of these impairments, allowing classification of patients according to the severity of PD.

After 190 spatiotemporal features were extracted from the wearable sensor system, the final feature sets were selected for the regression models and features were included from both upper and lower limbs, reinforcing the hypothesis that a complete motion analysis provides better results than using a reduced set of exercises.^5^

Indeed, the set of spatiotemporal features that obtained the maximum accuracy included parameters extracted from Gait, Heel-Toe Tapping, Pronation/Supination, Finger Tapping, Arms Swing, and Postural Tremor, confirming the significance of these exercises already found in previous works^4^,^5^ for the assessment of motor dysfunction in patients with PD. Furthermore, the selected features are derived from accelerometers and gyroscopes, suggesting that both sensors are equally important for capturing clinically relevant information.

Among the different investigated feature selection methods, the “*CfsSubsetEval*” provided the best solution for selecting a feature array of 13 parameters to use as input for the regression algorithms. In particular, the best performance was obtained with the ANFIS model that reached a correlation coefficient equal to 0.814. The good results endorse the significance of the selected parameters to evaluate the motor performance in PD patients. ANFIS and SVR were also investigated together previously for the assessment of the PD progression,^11^,^12^,^17^ and particularly ANFIS already achieved the highest accuracy^12^,^17^ for the assessment of disease progression, promoting the idea that such algorithm could represent a robust method to assess the progressive degenerative process that characterizes the PD development. Thus, it should be further investigated in future studies using larger datasets.

Since ANFIS is a combination of a fuzzy inference system (FIS) and neural network (NN), it can learn automatically from the data, which incidentally is the strength of feed-forward artificial NN.^17^ NN represents a broad class of computational models inspired by biological networks found in the central nervous systems and animal brains. They can be used to approximate unknown mapping of a large number of inputs,^12^ such as the parameters derived from the wearable system used in this study. Moreover, the C-means clustering used to develop the ANFIS model improved the predictive accuracy. Fuzzy C-means is a method of clustering that allows one piece of data to belong to two or more clusters. This capability is important for the intended application since the measurements used for PD assessment include several nonlinear patterns.

Nevertheless, the main limitation of the ANFIS model is that it cannot work very well with many features. Thus, there is the need to identify a reduced number of significant features that allows the highest accuracy to be obtained when using this predictor method.

Additionally, even if a sizeable number of subjects were involved in the study when considering different stages of pathology, the size of each group drastically decreases, resulting also in unbalanced groups that is a limitation of this study and could bias the accuracy of the system. Thus, in future works, the dataset should be enlarged, paying attention to recruiting balanced groups of patients with different levels of the disease. Also, in this work we had not the possibility to average multiple clinical gradings from different physicians, but we performed single evaluations on patients collaborating with the Neurology department of the Apuane Hospital, a small hospital in the Nord-West area of Tuscany, Italy. The Neurology department contributed to this study with a team, composed of two neurologists (the head physician and another physician) and one assistant physician that performed all the clinical evaluations. Adding more clinicians for evaluating the performance of the patients according to the clinical scales could reduce the inter-rater variability and improve the accuracy of the machine learning methods. In future works, we are managing multicenter studies with HD video recording evaluations to enable more physicians to clinically evaluate patients directly in place or offline later and then make a more statistically robust grading and data processing.

In conclusion, this work shows a high linear correlation between the extracted information from the sensors and clinical scores provided by the clinician. Therefore, this work represents a first unavoidable step toward a data-driven objective assessment of the pathology that could support the neurologist in improving the accuracy of the PD evaluations. Such a system, combining wearable technologies and artificial intelligence techniques, can aim at overcoming the traditional assessment of the pathology, based on semi-quantitative scales with a limited number of levels, where the scores are mainly based on the expertise of the clinicians, and provide a more continuous grading of the disease, defined on the basis of objectively measured parameters. Certainly, a data-driven assessment can help to reduce the inter-rater variability that typically affects the PD diagnosis, moving to more precise evaluations, and potentially improve the quality of care for PD patients.

## Electronic supplementary material

Below is the link to the electronic supplementary material.

Supplementary material 1 (MP4 3932 kb)
